# Arthroscopic Capsular Plication Shows Low Failure Rates and Meaningful Functional Improvement in Multidirectional Shoulder Instability With and Without Hypermobile Ehlers‐Danlos Syndrome

**DOI:** 10.1002/ars2.70077

**Published:** 2026-09-09

**Authors:** Chandler M. Catanzaro, Caleb W. Rogers, Joseph A. Q. Karam, Mitchell H. Negus, Brandon L. Rogalski, Richard J. Friedman, Josef K. Eichinger

**Affiliations:** ^1^ Department of Orthopaedics and Physical Medicine Medical University of South Carolina Charleston South Carolina U.S.A.

## Abstract

**Purpose:**

To evaluate clinical outcomes and failure rates after arthroscopic capsular plication for multidirectional shoulder instability in patients with and without hypermobile Ehlers‐Danlos syndrome.

**Methods:**

A retrospective case series with prospectively collected outcomes was conducted between 2015 and 2023 in patients undergoing arthroscopic capsular plication for multidirectional shoulder instability with and without hypermobile Ehlers‐Danlos syndrome, with a minimum of 2 years of follow‐up. Outcomes included American Shoulder and Elbow Surgeons, Single Assessment Numeric Evaluation, and Visual Analog Score pain scores. Failure was defined as recurrent instability or revision surgery.

**Results:**

Patients showed significant improvement in functional outcome scores and pain after surgery. Failure occurred in 6.7% of patients at a mean of 17 months postoperatively. Clinically meaningful improvement exceeding the minimal clinically important difference was achieved by 100% of patients for American Shoulder and Elbow Surgeons, 65.2% for Single Assessment Numeric Evaluation, and 64.5% for Visual Analog Score pain.

**Conclusions:**

Arthroscopic capsular plication was associated with meaningful functional improvement and a low failure rate in patients with multidirectional shoulder instability with and without hypermobile Ehlers‐Danlos syndrome.

**Level of Evidence:**

Level IV, therapeutic‐retrospective case series.

## Graphical abstract

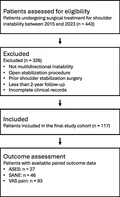

Hypermobile Ehlers‐Danlos syndrome (hEDS) is a connective tissue disorder defined by joint hypermobility leading to recurrent instability and an increased risk of dislocations and subluxations.^1^ The primary cause of hEDS is a defect in healthy collagen production resulting in a high degree of soft tissue laxity and poor mechanical stability.^2^ Shoulder instability in this patient population poses a significant challenge for physicians to treat, as conservative management often fails leading to the need for surgical intervention.^1^ Because of collagen deficiencies and the higher risk of soft tissue deformation, surgical outcomes remain variable, with reported failure rates that are higher than those in nonhyperlax populations.^1^ Multidirectional instability (MDI), which may occur independently or in association with hEDS, represents a particularly challenging subset of shoulder instability because of generalized capsular laxity and impaired static stabilization.

Surgical techniques have been employed to treat and manage shoulder instability in hyperlax patients; however, the effectiveness of these techniques in hEDS remains uncertain. The open capsular shift, introduced by Neer and Foster in 1980,^3^ aims to reduce instability by tightening the capsule and decreasing joint volume. This improves static stabilization and maintains articular integrity. Despite its success, this open procedure requires more soft tissue dissection, which can lead to longer recovery times and an increased degree of morbidity. Less invasive alternatives, such as arthroscopic techniques like capsular plication, have gained traction especially for patients with general laxity. Witney‐Lagen et al. investigated arthroscopic plication in patients with MDI, including those with hyperlaxity, and discovered that while many patients had improved stability, failure rates remained high.^4^ These findings highlight the challenges of surgical management in hyperlax patients and emphasize the need for continued evaluation of optimized surgical techniques.

Arthroscopic capsular plication has been introduced as a minimally invasive alternative to the open procedure, with advantages such as relatively preserved anatomy, lower morbidity, and higher visualization of the anterior and posterior labral complexes.^5^ Housset et al. report that arthroscopic capsular plication maintains labrum and cartilage integrity, making capsular adjustments easier. Sahin et al.^6^ back this report and discuss other various benefits like reduced soft tissue disruption, smaller incisions, less postoperative pain, and quicker recovery. A systematic review by Rolfes et al. found that arthroscopic capsular plication boasted a success rate of 91% in hyperlax patients, suggesting that it is a realistic alternative to the traditional open procedure in this patient population.^7^ Despite these advantages, surgical failure remains a concern in hyperlax populations, including patients with hEDS. Homere et al.^8^ conducted a systematic review of orthopaedic surgeries in hEDS patients, reporting high instability recurrence rates even with surgical intervention, underscoring the need for individualized strategies. Additionally, Broida et al. highlighted the importance of choosing appropriate surgical techniques for hEDS, emphasizing that these patients’ relative soft tissue fragility complicates achieving stable outcomes, reinforcing the need for further research and tailored surgical strategies.

The purpose of this study was to evaluate clinical outcomes and failure rates after arthroscopic capsular plication for MDI in patients with and without hEDS. We hypothesized that arthroscopic capsular plication would result in significant functional improvement with low failure rates in patients with and without hEDS.

## METHODS

A retrospective case series with prospectively collected outcomes was conducted on patients who underwent arthroscopic capsular plication for shoulder instability between 2015 and 2023 at a single academic institution.

Patients were included if they had a diagnosis of MDI, with or without hEDS, and had a minimum of 2 years of clinical follow‐up after arthroscopic capsular plication. Patients were excluded if they did not meet diagnostic criteria for MDI, underwent alternative stabilization procedures, or lacked a minimum of 2 years of postoperative follow‐up. A strengthening the reporting of observational studies style flow diagram detailing patient identification, inclusion, and exclusion is provided in Figure 1.

**FIGURE 1:**
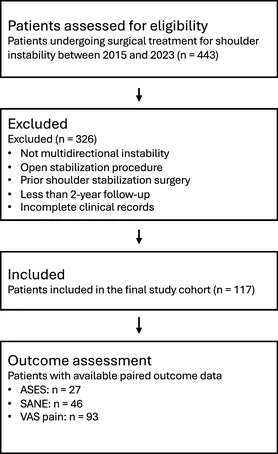
STROBE‐style flow diagram showing patient identification, application of inclusion and exclusion criteria, final study cohort inclusion, and availability of paired outcome data. Patients undergoing surgical treatment for shoulder instability were assessed for eligibility, with exclusions applied for non‐multidirectional instability diagnoses, alternative stabilization procedures, prior shoulder stabilization surgery, insufficient follow‐up, or incomplete clinical records. The final analytic cohort included 117 patients. Availability of paired preoperative and postoperative outcome measures varied by outcome, accounting for differences in sample sizes for ASES, SANE, and VAS analyses. (ASES, American Shoulder and Elbow Surgeons; SANE, Single Assessment Numeric Evaluation; STROBE, strengthening the reporting of observational studies; VAS, Visual Analog Score.)

Patients were identified retrospectively through a review of institutional surgical databases and electronic medical records. Eligible patients included those who underwent arthroscopic capsular plication for MDI during the study period. Outcomes were collected prospectively as part of routine clinical follow‐up and supplemented by a retrospective chart review when necessary.

MDI was diagnosed clinically based on symptomatic instability in at least 2 directions (anterior, posterior, or inferior), supported by physical examination findings, including a positive sulcus sign and symptomatic translation on load‐and‐shift testing. The diagnosis of hEDS was based on prior clinical diagnosis documented in the medical record in accordance with the 2017 international diagnostic criteria, including generalized joint hypermobility as assessed by the Beighton score and associated systemic features.

Electronic medical records were reviewed to gather demographic information, clinical characteristics, surgical details, and functional outcomes. Patient‐reported outcome measures, including American Shoulder and Elbow Surgeons (ASES), Single Assessment Numeric Evaluation (SANE), and Visual Analog Score (VAS) scores, were collected during routine clinical follow‐up and supplemented by direct patient outreach when necessary. Institutional review board (IRB) approval for this study was obtained through the Medical University of South Carolina (Pro00147312).

Data collected included patient demographics such as age at surgery and sex, Beighton scores, surgical variables including anchor configuration (anterior or posterior labrum vs anterior and posterior labrum), duration of postoperative immobilization (in weeks), and remplissage technique (yes/no). Outcome measures analyzed included preoperative and postoperative ASES, SANE, and VAS pain scores.

All procedures were performed by 1 of 5 fellowship‐trained surgeons (J.K.E., R.J.F., S.W., H.S., E.E.). All procedures were performed arthroscopically with the patient in the lateral decubitus position under general anesthesia. Standard posterior and anterior working portals were used. Diagnostic arthroscopy was performed to assess capsulolabral pathology, capsular laxity, and associated intra‐articular lesions. Capsular plication was performed using suture anchors placed along the glenoid rim to reduce the capsular volume and restore stability. The number of suture anchors used was determined intraoperatively based on the degree of capsular laxity, direction of instability, glenoid size, and surgeon judgment. Greater degrees of MDI and capsular redundancy generally warranted a higher number of fixation points to achieve adequate capsular tensioning. Posterior anchor placement was performed in cases with clinically or arthroscopically evident posterior instability or capsular redundancy, as determined by preoperative examination and intraoperative assessment.

Remplissage was performed selectively in the presence of engaging or clinically significant Hill‐Sachs lesions felt to contribute to instability, based on surgeon assessment. Nonengaging lesions were managed without remplissage.

Postoperatively, patients were immobilized in a sling for a duration determined by surgeon preference and perceived stability, typically ranging from less than 4 weeks to 4 weeks or longer. Progressive rehabilitation was initiated thereafter according to standardized institutional protocols.

Patient‐reported outcome measures included the ASES score, SANE score, and VAS for pain. Preoperative scores were collected at the initial clinical evaluation. Postoperative outcome measures were obtained at the most recent clinical follow‐up, with a minimum follow‐up duration of 2 years. Clinical follow‐up consisted of routine postoperative visits, with outcome measures collected during in‐person evaluations or via standardized questionnaires administered at the final follow‐up. Failure was defined as recurrent shoulder instability after surgery, including documented dislocation, symptomatic subluxation events reported by the patient, or the need for revision stabilization surgery. Time to failure was calculated from the date of index surgery to the first documented instability event or revision procedure.

Patients were then stratified for exploratory analysis by anchor configuration (anterior or posterior labrum vs anterior and posterior labrum), immobilization duration (<4 weeks vs ≥4 weeks), Beighton score (<5 vs ≥5), and remplissage status (yes vs no) for descriptive subgroup comparison.

The cutoff of 4 weeks for immobilization duration was selected based on the consensus rehabilitation guideline published by the American Society of Shoulder and Elbow Therapists, which recommends a variable period of absolute immobilization between 0 and 4 weeks after capsulolabral repair with suture or suture anchor fixation.

The choice of a Beighton score cutoff of 5 is justified by the evidence‐based consensus for adults up to age 50, as outlined in the GeneReviews criteria, which recommend a threshold of 5 or greater to define generalized joint hypermobility in this age group.

Statistics were calculated for all variables using univariate analyses. Because of the non‐normal distribution of data, the Mann‐Whitney U test was used for unpaired comparisons between groups. Mean differences were reported for each cohort. Continuous variables were rounded to a clinically appropriate precision. A *P* value of <.05 was considered statistically significant. All statistical analyses, tables, and figures were performed using GraphPad Prism software (version 10.4.2). Minimal clinically important difference (MCID) thresholds were calculated for each outcome measure using one‐half the standard deviation of the change scores. MCID achievement was assessed at the individual patient level and reported as the proportion of patients meeting or exceeding the MCID threshold for each outcome. No multivariable regression analysis was performed because of the limited number of failure events.

## RESULTS

A total of 117 patients undergoing arthroscopic capsulorrhaphy for shoulder instability related to hEDS, MDI, or both were included in the study (Figure 1). Of the cohort, 7.7% had a diagnosis of hEDS alone, 69.2% of patients had a diagnosis of MDI alone, and 23.1% of the cohort had a diagnosis of hEDS plus MDI. The cohort consisted of 65.0% female and 35.0% male patients, with a mean age of 27.2 ± 10.8 years. The mean preoperative Beighton score was 6.2 ± 1.9. Table 1 summarizes the baseline demographic and clinical characteristics of the cohort.

**TABLE 1:** Baseline Demographics and Clinical Characteristics

Characteristic	Value
Total patients (n)	117
Sex, n (%)	
Female	76 (65.0)
Male	41 (35.0)
Age, yr, mean ± SD (range)	27.2 ± 10.8 (51)
BMI, mean ± SD (range)	27.2 ± 7.3 (43)
Beighton score, mean ± SD (range)	6.2 ± 1.9 (8)
Diagnosis, n (%)	
hEDS alone	9 (7.7%)
MDI alone	81 (69.2%)
hEDS + MDI	27 (23.1%)

BMI, body mass index; hEDS, Hypermobile Ehlers‐Danlos Syndrome; MDI, multidirectional instability; SD, standard deviation; yr, year.

A significant improvement was observed across all outcome measures after surgery. The mean preoperative ASES score was 47.0 ± 17.7 which improved to 93.6 ± 6.9 postoperatively (*P* < .0001, n = 27, paired t‐test) (Figure 2A). Similarly, mean SANE scores improved from 45.0 ± 20.0 preoperatively to 84.8 ± 15.5 postoperatively (*P* < .0001, n = 46) (Figure 2B). VAS pain scores significantly decreased from a mean of 4.4 ± 2.5 preoperatively to 1.6 ± 2.2 postoperatively (*P* < .0001, n = 93) (Figure 2C). Not all patients had complete paired preoperative and postoperative patient‐reported outcome measures available at final follow‐up; therefore, analyses were performed using available paired data for each outcome measure. Because the availability of paired outcome data differed by measure, subgroup sample sizes varied by outcome.

**FIGURE 2:**
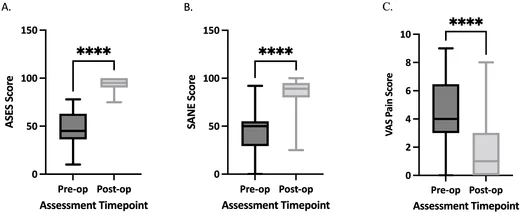
Preoperative and postoperative (A) ASES, (B) SANE, and (C) VAS pain scores after arthroscopic capsular plication. Box‐and‐whisker plots display the median (center line), interquartile range (box), and range (whiskers). Paired comparisons were performed using paired statistical testing. **** indicates *P* < .0001. Sample sizes reflect available paired outcome data (ASES, n = 27; SANE, n = 46; VAS, n = 93). (ASES, American Shoulder and Elbow Surgeons; Post‐op, postoperative; Pre‐op, Preoperative; SANE, Single Assessment Numeric Evaluation; VAS, Visual Analog Score.)

Using cohort‐specific MCID thresholds, clinically meaningful improvement was achieved by 27 of 27 patients (100%) for ASES, 30 of 46 patients (65.2%) for SANE, and 60 of 93 patients (64.5%) for VAS pain.

Among patients with available paired outcome data, when stratified by anchor configuration, there were no statistically significant differences in postoperative outcomes between patients treated with anchors in an anterior or posterior labrum configuration versus an anterior and posterior labrum configuration. However, general improvements were observed among all postoperative outcome score categories. Specifically, mean improvements in ASES scores were similar between groups (42.9 ± 18.1 vs 46.0 ± 19.1; *P* = .6951, n = 20) as were SANE scores (33.5 ± 23.6 vs 41.1 ± 22.8; *P* = .3346, n = 40) and VAS pain scores (−2.5 ± 2.8 vs −2.8 ± 2.5; *P* = .4044, n = 89) (Table 2). These findings suggest that anchor configuration alone did not significantly influence functional or pain‐related outcomes after arthroscopic capsulorrhaphy in this cohort.

**TABLE 2:** Postoperative Improvements Stratified

Variable	ΔASES	ΔSANE	ΔVAS
Beighton score			
<5	25.9 ± 11.1 (n = 4)	31.7 ± 20.7 (n = 6)	−1.8 ± 1.4 (n = 12)
≥5	24.8 ± 4.1 (n = 8)	43.0 ± 21.0 (n = 8)	−3.4 ± 1.9 (n = 20)
*P* value	.83	.19	.916
Immobilization, wks			
<4	51.8 ± 14.3 (n = 11)	46.1 ± 23.1 (n = 14)	−3.0 ± 2.9 (n = 36)
≥4	43.0 ± 19.9 (n = 16)	32.3 ± 22.1 (n = 30)	−2.5 ± 2.4 (n = 57)
*P* value	.631	.238	.783
Anchor configuration			
Anterior or posterior labrum	42.9 ± 18.1 (n = 8)	33.5 ± 23.6 (n = 14)	−2.5 ± 2.8 (n = 35)
Anterior and posterior labrum	46.0 ± 19.1 (n = 12)	41.1 ± 22.8 (n = 26)	−2.8 ± 2.5 (n = 55)
*P* value	.6951	.3346	.4044
Remplissage			
Used		51.1 ± 13.7 (n = 8)	
Not used		37.4 ± 23.2 (n = 38)	
*P* value		.2717	

*Note*: Subgroup totals may exceed or differ from overall subgroup counts because of outcome‐specific availability of paired data. All values are reported as mean ± SD. Sample sizes reflect patients with available paired preoperative and postoperative outcome data for each subgroup and outcome measure; therefore, n varies by outcome. Beighton score is assessed using the standard 9‐point scale. ΔASES and ΔVAS were not analyzed for the remplissage subgroup because of insufficient paired outcome data.

ASES, American Shoulder and Elbow Surgeons; SANE, Single Assessment Numeric Evaluation; SD, standard deviation; VAS, Visual Analog Scale; wks, weeks.

Assessment by duration of postoperative immobilization (<4 weeks vs ≥4 weeks) also revealed no significant differences in ASES (51.8 ± 14.3 vs 43.0 ± 19.9; *P* = .631, n = 27), SANE (46.1 ± 23.1 vs 32.3 ± 22.1; *P* = 0.238, n = 44), or VAS pain scores (−3.0 ± 2.9 vs −2.5 ± 2.4; *P* = 0.783, n = 90) (Table 2).

No significant differences were found when comparing outcomes between patients with Beighton scores <5 vs ≥5. ASES (25.9 ± 11.1 vs 24.8 ± 4.1; *P* = 0.83, n = 12), SANE (31.7 ± 20.7 vs 43.0 ± 21.0; *P* = 0.19, n = 14), and VAS (−1.8 ± 1.4 vs −3.4 ± 1.9; *P* = 0.916, n = 32) (Table 2) score improvements were comparable between these subgroups.

SANE scores improved in both remplissage and no‐remplissage groups, with no significant difference between them (51.1 ± 13.7 vs 37.4 ± 23.2; *P* = 0.2717, n = 46) (Figure 2).

Failure, defined as recurrent instability or a revision procedure, occurred in 8 patients (6.7%). The mean time to failure was 17.4 ± 10.7 months (Table 3). Failure occurred in 6 of 76 female patients (7.9%) and 2 of 41 male patients (4.9%). Given the small number of failure events, no formal statistical comparison by sex was performed. Failures consisted of recurrent instability events, including symptomatic subluxation or dislocation and/or revision stabilization surgery.

**TABLE 3:** Postoperative Failure Rate

Variable	Value
Total patients (n)	117
Postoperative failure, n (%)	8 (6.7)
Time to failure, months, mean ± SD	17.4 ± 10.7

SD, standard deviation.

## DISCUSSION

Arthroscopic capsular plication for shoulder instability in patients with hEDS and/or MDI resulted in significant improvements in functional outcomes and pain scores, with a low rate of recurrent instability at midterm follow‐up. These findings support arthroscopic capsular plication as a viable surgical option for shoulder instability in hypermobile and/or hyperlax patients while highlighting the need for individualized surgical decision‐making and rehabilitation strategies.

Patients showed statistically and clinically significant improvements in functional outcome scores and pain after surgery. These improvements reinforce the clinical relevance of surgical intervention in this patient population. Similar improvements have been noted in prior studies investigating arthroscopic surgeries in MDI patients, including those with connective tissue disorders, where postoperative ASES scores higher than 85 have been reported.^9^, ^10^, ^11^

Our findings align with prior literature showing that arthroscopic stabilization can be effective in hyperlax and hEDS populations, though outcomes remain variable. Like Witney‐Lagen et al., who reported improved stability but high rates of persistent failure after arthroscopic plication in MDI patients,^4^ our study observed significant functional gains with a relatively low failure rate (6.7%). This compares favorably with earlier arthroscopic series by Gartsman et al. and Baker et al., who reported failure rates ranging from 9% to 17% in MDI cohorts.^9^, ^12^ Additionally, our postoperative ASES and SANE scores mirror those reported by Alpert et al. and Jacobson et al., who found durable improvements after arthroscopic and open stabilization for MDI.^8^, ^13^ In contrast, older interventions such as thermal and laser capsulorrhaphy showed higher recurrence rates and less predictable outcomes, underscoring the superiority of current arthroscopic techniques.^14^, ^15^ Importantly, our finding that the Beighton score did not predict outcomes is consistent with Sahu et al., who showed that shoulder hyperlaxity did not adversely affect recurrence after the Latarjet procedure.^16^ Conversely, Vavken et al. noted higher recurrence rates in adolescents with generalized hyperlaxity treated with open inferior capsular shift,^17^ suggesting that age and tissue quality may modulate the relation between hypermobility and surgical durability. Collectively, these findings suggest that with modern arthroscopic techniques and individualized rehabilitation, outcomes in hyperlax populations may approach those seen in nonhyperlax cohorts.

No statistically significant differences were observed between anchor configurations, suggesting that the fixation strategy alone did not meaningfully influence postoperative outcomes in this cohort. These findings indicate that while secure anchor placement remains important, no specific anchor configuration was associated with superior postoperative outcomes within this cohort. Prior biomechanical studies have reported that anterior and posterior labrum suture placement may improve joint constraint, but our data does not support superior functional outcomes.^11^, ^18^, ^19^

The duration of postoperative immobilization was not associated with significant differences in functional or pain‐related outcomes. Prior literature has explored the role of early controlled motion after shoulder stabilization; however, the present findings should be interpreted cautiously given the nonrandomized nature of postoperative rehabilitation.^20^, ^21^

The Beighton score did not significantly influence postoperative outcome improvements, consistent with prior studies examining generalized hypermobility.^16^ Accordingly, these subgroup findings should be interpreted cautiously and are intended to be hypothesis‐generating rather than definitive. As such, these subgroup findings should not be interpreted as evidence of equivalence between techniques but rather as descriptive observations within the limitations of the study design.

The overall failure rate was 6.7%, defined by either recurrent instability or need for further intervention, with a mean time of failure of 17.35 ± 10.68 months. This rate compares favorably to MDI and instability patient populations at large, where failure rates have been reported to fall within the 7% to 17% range.^22^, ^23^, ^24^ Our data suggest that outcomes in patients with hEDS and MDI may approach those reported in non‐hypermobile patient populations when managed with contemporary arthroscopic stabilization techniques.

Although our initial hypothesis proposed that anchor configuration, immobilization duration, and Beighton score would influence postoperative durability, subgroup analyses did not show statistically significant differences across these variables. These findings should be interpreted as exploratory, as treatment selection was guided by clinical judgment and subgroup analyses were not adjusted for potential confounding factors. It is possible that unmeasured variables, including intraoperative capsular tensioning and postoperative rehabilitation adherence, play a more substantial role in determining outcomes.

Future studies should evaluate the relation between hypermobility, capsular tensioning, and dynamic stability. There is a need for randomized trials comparing early versus delayed physical rehabilitation in hypermobile patients. This could clarify whether guided, controlled early motion improves long‐term function in this patient population. Larger, multicenter cohorts may provide the power needed to detect more subtle subgroup differences in high‐risk patient populations like those with connective tissue disorders.

### Limitations

This study is limited by its retrospective design and the lack of a randomized or matched control group. Our analysis is subject to limitations including selection bias and the inability to control for confounding variables. Although the overall sample size was adequate, subgroup analyses were exploratory and underpowered, limiting the ability to detect subtle differences. Postoperative rehabilitation strategies were not standardized or recorded in sufficient detail across all patients. Furthermore, as a single‐center study performed by a limited number of surgeons, our results may not be fully generalizable to other settings. Not all patients provided paired preoperative and postoperative patient‐reported outcome measures, which may introduce response bias. These limitations should be considered when interpreting the findings of this study. The study was underpowered to detect sex‐based differences in failure rates. A Kaplan‐Meier survival analysis was not performed because of the small number of failure events, which would limit the interpretability of survival estimates.

## CONCLUSIONS

Arthroscopic capsular plication was associated with meaningful functional improvement and a low failure rate in patients with multidirectional shoulder instability with and without hEDS.

## DISCLOSURES

The authors (R.J.F., J.K.E.) declare the following financial interests/personal relationships which may be considered as potential competing interests: R.J.F. receives grants or contracts and royalties or licenses from Exactech. J.K.E. receives grants or contracts from Exactech and serves in a leadership or fiduciary role in other board, society, committee, or advocacy group, paid or unpaid, as a member of the Editorial Board of the Journal of *Arthroscopy*. The other authors (C.M.C., C.W.R., J.A.Q.K., M.H.N., B.L.R.) declare that they have no known competing financial interests or personal relationships that could have appeared to influence the work reported in this article.
